# Autoimmune Lymphoproliferative Syndrome-FAS Patients Have an Abnormal Regulatory T Cell (Treg) Phenotype but Display Normal Natural Treg-Suppressive Function on T Cell Proliferation

**DOI:** 10.3389/fimmu.2018.00718

**Published:** 2018-04-09

**Authors:** Fabienne Mazerolles, Marie-Claude Stolzenberg, Olivier Pelle, Capucine Picard, Benedicte Neven, Alain Fischer, Aude Magerus-Chatinet, Frederic Rieux-Laucat

**Affiliations:** ^1^INSERM UMR1163, Laboratory of Immunogenetics of Paediatric Autoimmunity, Paris, France; ^2^Paris Descartes—Sorbonne Paris Cité University, Imagine Institute Paris, Paris, France; ^3^INSERM UMR1163, Cell Sorting Facility, Paris, France; ^4^Paediatric Haematology-Immunology and Rheumatology Unit, Necker-Enfants Malades Hospital, Assistance Publique—Hôpitaux de Paris (APHP), Paris, France; ^5^Center for Primary Immunodeficiencies, Necker-Enfants Malades Hospital, APHP, Paris, France; ^6^Laboratory of Lymphocyte Activation and Susceptibility to EBV Infection, INSERM UMR 1163, Imagine Institute, University Paris Descartes Sorbonne Paris Cité, Paris, France; ^7^Collège de France, Paris, France

**Keywords:** human, autoimmune lymphoproliferative syndrome-FAS, cell proliferation, regulatory T cell, suppression assay

## Abstract

**Objective:**

Autoimmune lymphoproliferative syndrome (ALPS) with FAS mutation (ALPS-FAS) is a nonmalignant, noninfectious, lymphoproliferative disease with autoimmunity. Given the central role of natural regulatory T cells (nTregs) in the control of lymphoproliferation and autoimmunity, we assessed nTreg-suppressive function in 16 patients with ALPS-FAS.

**Results:**

The proportion of CD25^high^CD127^low^ Tregs was lower in ALPS-FAS patients than in healthy controls. This subset was correlated with a reduced CD25 expression in CD3^+^CD4^+^ T cells from ALPS patients and thus an abnormally low proportion of CD25^high^FOXP3^+^ Helios^+^ T cells. The ALPS patients also displayed a high proportion of naïve Treg (FOXP3^low^CD45RA^+^) and an unusual subpopulation (CD4^+^CD127^low^CD15s^+^CD45RA^+^). Despite this abnormal phenotype, the CD25^high^CD127^low^ Tregs’ suppressive function was unaffected. Furthermore, conventional T cells from *FAS*-mutated patients showed normal levels of sensitivity to Treg suppression.

**Conclusion:**

An abnormal Treg phenotype is observed in circulating lymphocytes of ALPS patients. However, these Tregs displayed a normal suppressive function on T effector proliferation *in vitro*. This is suggesting that lymphoproliferation observed in ALPS patients does not result from Tregs functional defect or T effector cells insensitivity to Tregs suppression.

## Introduction

Autoimmune lymphoproliferative syndrome (ALPS) is characterized by defective lymphocyte apoptosis and thus the dysregulation of lymphocyte homeostasis (1). In turn, this dysregulation leads to nonmalignant polyclonal lymphoproliferation and then splenomegaly, lymphadenopathy, and hepatomegaly. Autoimmune manifestations (mostly autoimmune hemolytic anemia, thrombocytopenia, and, in some cases, neutropenia) are observed in two-thirds of patients with ALPS (2). An elevated risk of lymphoma has also been reported. The characteristic laboratory abnormalities include the expansion of T cells that express the alpha/beta T cell receptor but lack both CD4 and CD8 in peripheral blood and tissue samples (i.e., double-negative (DN) T cells) (3). These DNT cells have been shown to originate from the terminally differentiated effector memory CD45RA^+^ (TEMRA) peripheral CD4^+^ or CD8^+^ T lymphocytes (4). Other laboratory findings include elevated serum levels of interleukin (IL)-10, vitamin B12, and soluble Fas ligand. ALPS is genetically heterogeneous, and both somatic and germline mutations have been identified in the *TNFRSF6* (encoding FAS and also referred to as *CD95* or *Apo-1*), *FAS-LG* (Fas ligand), and *CASP10* genes (5–8). Immunosuppressive treatments, including steroids or lymphotoxic drugs, are usually efficient to control the disease. Importantly, hyperactivation of the mTOR pathway has been shown to promote the lymphoproliferation in ALPS-FAS patients (9). This seminal study on ALPS pathophysiology prompted the clinicians to use mTOR-inhibitor-based treatments in ALPS patients, which showed very good efficacy (10). Mutations in *TNFRSF6* impair the formation of the death-inducing-signaling complex or the Fas/Fas ligand interaction (either by modifying Fas’ structure or precluding its membrane expression) (11). Thus, a defect in this pathway leads to the expansion of T and B lymphocytes including self-antigen-specific populations and thus autoimmunity as a consequence of cell death resistance. Indeed, a well-designed experiment, taking advantage of a loss-of-start mutation accompanied with somatic loss of heterozygosity, evidenced a disturbed B-lymphocytes selection in ALPS-FAS patients (12). Moreover, the role of the B-cell subset in the pathophysiology is also underlined by the increased risk of lymphoma, mostly of B-cell origin (13–15). However, there is no correlation between the magnitude of the apoptosis defect *in vitro* and the severity of the disease *in vivo*. Moreover, some *FAS* mutations may not be sufficient to trigger the disease, since asymptomatic carriers of germline *FAS* mutations have been described (i.e., partial clinical penetrance). The magnitude of the functional T cell defect is similar in asymptomatic carriers and symptomatic patients. Furthermore, ALPS is the only autoimmune syndrome in which a germline mutation on one *TNFRSF6* allele (72%) and a somatic mutation on the other (0.5%) leads to disease onset; this explains the observed clinical differences between carriers of heterozygous germline mutations. This accumulation of genetic events provides the mutated cells with a selective advantage and is thus analogous to Knudson’s two-hit hypothesis of carcinogenesis (16). This finding shows that somatic mutations can lead to autoimmune disease and might explain the incomplete penetrance observed in familial autoimmunity. Lastly, this finding implies the existence of factors that modify the onset of ALPS.

Along with programmed cell death, self-tolerance is also achieved *via* active suppression of lymphocyte proliferation by regulatory T cells (Tregs). The latter are defined phenotypically by the expression of CD4, FOXP3, and CD25 (the IL-2 receptor α chain) and the absence of CD127 (the IL-7 receptor) (17). Tregs have a key role in the prevention of autoimmunity and inflammation, as evidenced by the early-onset, severe autoimmune diseases caused by defects in Treg function or development (18, 19). Indeed, mutations of the human *FOXP3* gene (encoding the forkhead box P3 transcription factor) result in a fatal, systemic, autoimmune and inflammatory disease linked to the syndrome called immune dysregulation, polyendocrinopathy, enteropathy, X-linked (IPEX). CD25 deficiency also results in severe autoimmunity and allergy and is phenotypically indistinguishable from IPEX (20). IL-2 secretion by activated, conventional effector T cells (Tconvs) is critical for the development, survival, and function of FOXP3^+^ natural Tregs (nTregs) (21, 22). More recently, it has been suggested that the expression of Helios (an Ikaros family transcription factor that enhances FOXP3 expression by binding to the FOXP3 promoter (23) and represses the IL-2 gene promoter (24)) can be used to discriminate between (i) nTregs that differentiate in the thymus and (ii) induced Tregs (iTregs) or effector Treg (eTregs) that differentiate in the peripheral tissues following exposure to antigen (25). However, it has also been shown that the Helios^+^ and Helios^−^ nTregs have similar levels of suppressor activity and FOXP3 expression—suggesting that a lack of Helios expression is not a perfect marker of human iTregs (26).

Regulatory T cells suppress not only autoimmune responses but also other aberrant or excessive immune responses to non-self-antigens. There is now a growing body of evidence to suggest that Tregs can control almost all physiological or pathological responses of the adaptive immune system. Furthermore, several mechanisms of Treg-mediated suppression have been proposed; these include the secretion of immunosuppressive cytokines (IL-10 and TGF-β) and the cell–cell-contact-dependent suppression, functional modification, and killing of antigen-presenting cells (APCs). Alternatively, the absorption of cytokines by Tregs may deprive responder T cells of cytokines and thus induce apoptosis (27). The effectiveness of this suppression also depends on the sensitivity/resistance of Tconvs to the inhibitory effects of Tregs. We hypothesized that this key checkpoint for self-tolerance controls the lymphoproliferation and autoimmunity observed in ALPS-Fas. Hence, in order to check whether one or more functional defects in the Treg and/or Tconv populations might be involved in ALPS, we investigated the Tregs’ phenotype and function in ALPS patients with a Fas defect.

## Patients and Methods

### Cell Isolation

Purified PBMCs from adult CTs (French Blood Transfusion Service, Paris, France) and patients with ALPS were prepared by density gradient centrifugation on Lymphoprep (Abcyss SA). The donor age range was 21–64 years for CTs and 6–33 years for patients with ALPS. Tconvs, Tregs, and dendritic cells (DCs) were sorted using flow cytometry.

### Antibodies and Reagents

Anti-CD14 conjugated to violet blue was purchased from Miltenyi Biotec (Bergisch Gladbach, Germany). FITC-anti-CD4, PE Cy5-anti-CD11c, PE-anti-CD25, BV650-anti-CD25, APC-anti-CD45RA, BV711-anti-CD15s, PE-anti-CD45RA, PC5-anti-CD28, and PE anti-CTLA-4 were obtained from BD Biosciences (Mountain View, CA, USA). PE CY7 anti-CD127, BV421-anti CCR7, PE-anti-CD59, and BV510-anti-CD27 were purchased from Sony Biotechnology (San Jose, CA, USA); PE-FOXP3, APC-FOXP3, and eF450-Helios were supplied by eBiosciences (San Diego, CA, USA); PC5 anti-ICOS, APC-anti-PD1, BV421-anti-CD39, APC-anti-GITR, and BV421-anti LAG3 were purchased from Biolegend (San Diego, CA, USA); and staphylococcal enterotoxin E (SEE) was purchased from Toxin Technology Inc.; and CellTrace carboxyfluorescein succinimidyl ester (CFSE) was purchased from Molecular Probes (Eugene, OR, USA). The anti-CD3 (OKT3) used for some proliferation assays was purchased from Biolegend (San Diego, CA, USA).

### Purification of T Cells From PBMCs and Co-Culture With Sorted DCs

PBMCs were incubated for 30 min at 4°C with specific, labeled monoclonal antibodies, washed, and then sorted using an ARIA II cytometer (BD Biosciences). Naïve Tconvs were defined as CD4^+^CD25^−^CD127^+^CD45RA^+^ T cells, Tregs were defined as CD4^+^CD25^high^CD127^low^ T cells, nTregs were defined as CD4^+^CD25^high^CD127^low^CD45RA^+^ T cells, and memory Tregs were defined as CD4^+^CD25^high^CD127^low^CD45RA^−^ T cells. DCs were defined as CD11c^+^CD4^low^CD14^−^ cells and monocytes were defined as CD11c^+^CD4^low^CD14^+^ cells. After cell sorting, naïve Tconvs were washed and stained with CellTrace CFSE (to determine specific naïve Tconv proliferation). Next, the cells were washed and incubated with DCs (Tconv:sorted DC ratio of 1:0.4). Tregs were added, to give a Treg:naïve Tconv ratio of 1:0.2 or 1:1. SEE (0.2 ng/ml) was then added. The cell proliferation assay was performed with autologous and heterologous samples in Panserin medium (Dutscher, Brumath, France) supplemented with 5% human AB serum in 96-well plates. After 5 days of culture, the percentage of proliferating cells was measured with a MACSquant system (Miltenyi), and the data were analyzed with FlowJo software. Results were expressed as the percentage of proliferating cells. In the presence of Tregs or the results are expressed in percentage of the inhibition of proliferation. To test the reproducibility of these experiments, cells from the various patients were tested against different CTs or against the same CT. Similar results were obtained under both conditions.

A FOXP3 staining/permeabilizing buffer set (eBiosciences) was used for the intracellular staining of FOXP3, Helios, and CTLA-4.

### Patients and Ethical Aspects

We studied a cohort of 16 patients (summarized in Table 1) with typical manifestations of ALPS (adenopathy, lymphadenopathy, anemia, thrombopenia, and splenomegaly). The patients carried various heterozygous germline *TNFRSF6* mutations affecting the intracellular domain of the protein and were all registered in the French national primary immunodeficiency database (CEREDIH, Paris, France) (16). As indicated in Table 1, some patients were being treated with immunosuppressive treatments. Written informed consent (parental consent, in case of minors) was obtained from all participants of the study. The study protocols conform to the 1975 Declaration of Helsinki and were approved by the comité de protection des personnes Ile de France II and the French advisory committee on data processing in medical research.

**Table 1:** Clinical and genetic features of 16 ALPS patients.

Patients	Range age (years)	Germline FAS mutation	Somatic FAS mutation	Treatment	*In vitro* apoptosis defect	IL-10 (pg/ml)	FASL (ng/ml)	sCD25 (ng/ml)	DN/TCRab(%)
P1^a^	2–10		Ex9 : R250G	YES + C	NO	13	0.5	NT	2
P2	2–10	Ex9: D269fsX277		YES	/	110	4.3	NT	9
P3	2–10	Ex8:DelP217 X 220		YES	YES	51	0.3	>11	9
P4	2–10	Ex8:DelP217 X 220		NO	/	60	1.46	NT	21
P5	12–19	Ex 7: Q196X		YES	YES	90	1	>11	9
P6	12–19	Ex9: D265G		YES + C	YES	58	1	NT	7
P7	12–19	Ex7: E194fsx214		NO	YES	31	1.3	NT	14
P8	12–19	Ex9: S230fs X241		YES	YES	<1	0.4	>11	4
P9^a^	12–19		Ex 8 duplication	NO	NO	63	1.07	>11	29
P10^a^	12–19		Ex8:Del P217 X 220	NO	NO	101	1.2	NT	16
P11	20–29	Ex 9: G253D		YES	YES	20	1.1	9.3	14
P12^a^	20–29		Ex 8: del P217 fs X220	NO	NO	73	1.6	NT	10
P13^a^	20–29		Ex8: del- P217fs X220	YES	NO	203	4	NT	44
P14^a^	20–29	Ex 4: DelC135	IOH	YES	NO	38	0.74	NT	20
P15	>30		Ex9: D269 fsX 279	YES	YES	30	2.23	>11	19
P16	>30		Ex8:Del P217 X 220	NO	YES	1	0.34	NT	21

*The patients’ respective FAS mutations are indicated*.

*^a^Denotes somatic mutations*.

*Normative values are <20 pg/ml for IL-10, <0.2 ng/ml for FAS ligand, and <3% for the proportion of DN T lymphocytes*.

*In CTs, the serum level of sCD25 was around 2 ng/ml*.

*NT, not tested; IOH, loss of heterozygote; C, corticoid*.

### Statistical Analysis

All analyses were performed using GraphPad Prism software (version 6, GraphPad Software, Inc., La Jolla, CA, USA). A non-parametric Mann–Whitney *U*-test was used to compare the data for the various populations from the patients and controls.

## Results

### Phenotypic Characteristics of Tregs in ALPS Patients

**Figure 1:**
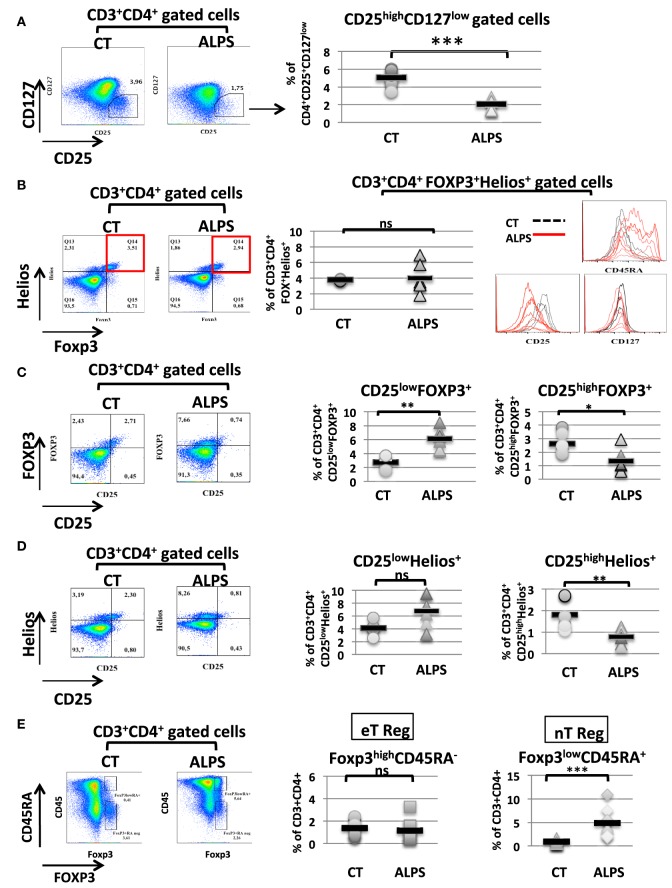
Phenotypic characteristics of regulatory T cells (Tregs) in autoimmune lymphoproliferative syndrome (ALPS) patients. Stained CD3^+^CD4^+^-gated T lymphocytes isolated from seven healthy controls (CTs) and seven ALPS patients (ALPS) are shown. **(A)** shows the mean ± SD (range) percentage of CD25^+^CD127^−^ CD3^+^CD4^+^ gated cells: 2.1 ± 0.2% (1.30–3.06%) in ALPS patients and 5.1 ± 0.2% (3.45–6.07%) in CTs (***p < 0.0001). **(B)** shows the mean ± SD (range) percentage of the FOXP3^+^Helios^+^ T cells subpopulation: 4 ± 1.7% (1.79–6.89%) in ALPS patients and 3.77 ± 0.1% (3.64–4%) in CTs. The histograms represent the staining of CD25, CD45RA, and CD127 on FOXP3^+^Helios^+^ CD3^+^CD4^+^-gated cells. For CTs (dashed black lines), the mean ± SD fluorescence intensities were 5,536 ± 1,275 (CD25), 1,518 ± 519 (CD45RA), and 280 ± 46 (CD127). For ALPS patients (solid red lines), the mean ± SD fluorescence intensities were 2,388 ± 658 (CD25), 7,171 ± 3,507 (CD45RA), and 197 ± 81 (CD127). In **(C)**, the mean ± SD (range) percentage of CD25^high^FOXP3^+^ T cells subpopulation from gated CD3^+^CD4^+^ T cells was 1.33 ± 0.8% (0.56–2.94%) in ALPS patients and 2.75 ± 0.75% (1.9–3.88%) in CTs. The percentage of CD25^low^FOXP3^+^ T cells is 6.1 ± 0.62% (4.41–8.42) in ALPS patients and 2.5 ± 0.38% (1.6–3.71) in CT (**p = 0.0022, *p = 0.026). In **(D)**, the mean ± SD (range) percentage of CD25^high^Helios^+^ T cells subpopulation was 0.78 ± 0.33% (0.32–1.25%) in ALPS patients and 2 ± 0.6% (1.3–2.7%) in CTs. The percentage of CD25^low^Helios^+^ T cells was 6.7 ± 2.3% (3.14–9.48%) in ALPS patients and 4.1 ± 1% (2.54–5.7%) in CTs (**p = 0.0043, ns: not significant). In **(E)**, the percentages of CD4^+^CD3^+^FOXP3^low^CD45RA^+^ nTregs and CD4^+^CD3^+^FOXP3^high^CD45RA^neg^ eTregs among PBMCs from ALPS patients (ALPS) are compared with the values for CTs (CONTROL). The error bars correspond to the mean ± SD for duplicates. The mean ± SD (range) percentage of eTregs was 1.36 ± 0.17% (0.81–2.39%) in CTs (*n* = 12) and 1.19 ± 0.3% (0.41–3.25%) in ALPS patients (*n* = 9). The mean ± SD (range) percentage of natural Tregs was 0.87 ± 0.12% (0.46–1.64%) in CTs (*n* = 12) and 4.9 ± 0.97% (1.83–10.90%) in ALPS patients (*n* = 9). Statistical analyses were performed using a non-parametric Mann–Whitney *U*-test (***p < 0.0001). ns: not significant.

**Figure 2:**
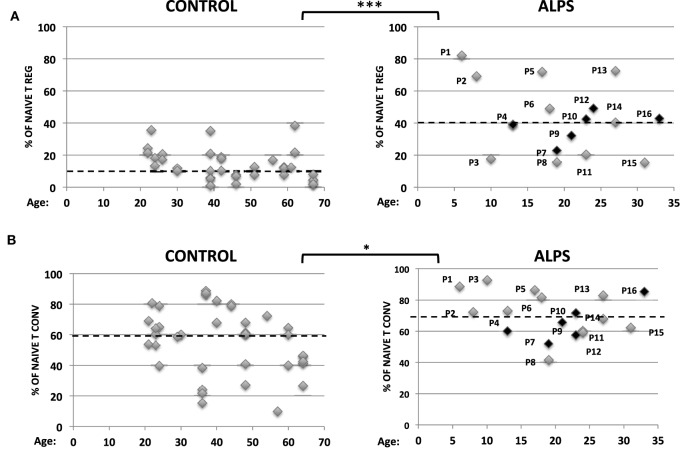
Proportions of naïve regulatory T cells (Tregs) and naïve conventional effector T cell (Tconvs) in autoimmune lymphoproliferative syndrome (ALPS) patients. Percentages of CD4^+^CD25^+^CD127^−^CD45RA^+^ naïve Tregs **(A)** and CD4^+^CD25^−^CD127^+^CD45RA^+^ naïve Tconvs **(B)** among PBMCs from 16 ALPS patients (right-hand panels) and healthy controls (CTs) (left-hand panels). The year of birth is indicated. For ALPS patients, the year of onset ranged from 2009 to 2014. Patients (P) not having been treated at the time of the assay are indicated in black. The hatched line represents the mean percentage of naïve T cell subpopulations. In panel A, the mean ± SD (range) percentage of naïve Treg was 14.7 ± 1.6% (2–38.3%) for CTs (*n* = 25) and 35.3 ± 4.5% (15–82%) for ALPS patients (*n* = 16). In panel B, the mean ± SD (range) percentage of naïve Tconvs was 56.5 ± 3.3% (10–88.8%) for CTs (*n* = 35) and 70 ± 3.3% (41–92.6%) for ALPS patients (*n* = 16). Statistical analyses were performed using a non-parametric Mann–Whitney *U*-test (***p < 0.0001; *p = 0.0205).

**Figure 3:**
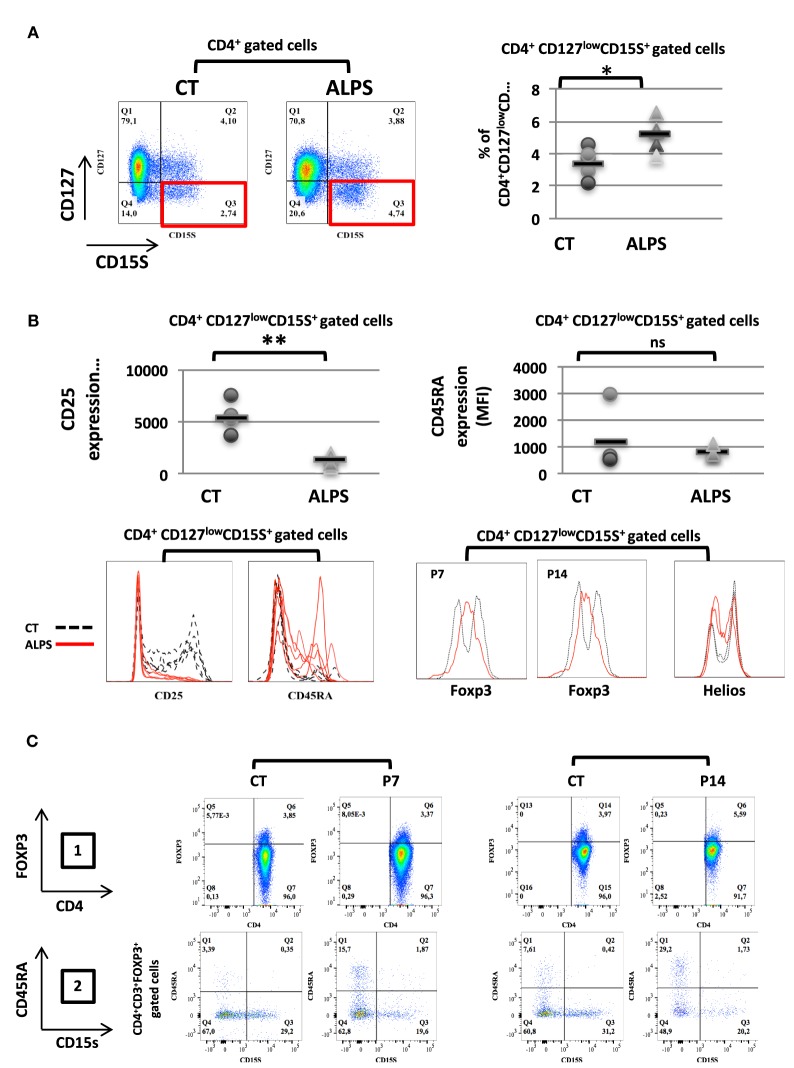
Proportion and phenotype of CD127^low^ CD15s^+^ regulatory T cell (Treg) subpopulations in autoimmune lymphoproliferative syndrome (ALPS) patients. **(A)** CD3^+^CD4^+^-gated T lymphocytes isolated from four healthy controls (CTs) or five ALPS patients (ALPS) were stained for CD15S and CD127. The mean ± SD (range) percentage of CD127^low^CD15s^+^ T cells subpopulation was 5.1 ± 0.4% (3.75–6.51%) in ALPS patients and 3.3 ± 0.4% (2.01–4.4%) in CTs is shown. **(B)** CD4^+^CD127^low^CD15s^+^-gated T cell subpopulations were stained for CD25, CD45RA, FOXP3, and Helios. The staining of FOXP3 and Helios was shown for two patients (P7 and P14) and two of the four CTs (left panel). The results for CD25, CD45RA in the CD4^+^CD127^low^CD15s^+^ T cell subpopulation are shown. For CTs (dashed black lines), the mean ± SD fluorescence intensities were 6,011 ± 1,950 (CD25) and 1,225 ± 1,048 (CD45RA). For ALPS patients (solid red lines), the mean ± SD fluorescence intensities were 1,299 ± 491 (CD25) and 761 ± 164 (CD45RA). (**p = 0.0029, *p = 0.0124, ns: not significant). **(C)** The expression of CD4 and FOXP3 was shown on CD3^+^CD4^+^-gated cells for two CTs, and two patients (P7 and P14) are shown in panel 1. The percentage of CD15s^+^CD45RA^neg^ cells on CD3^+^CD4^+^FOXP3^+^-gated cells is shown for two patients (P7 and P14) and two controls (CT) (panel 2). Statistical analyses were performed using a non-parametric Mann–Whitney *U*-test.

We studied 16 ALPS patients (Table 1). By studying the CD25 and CD127 surface markers, we observed abnormally low percentages of the CD25^high^CD127^low^ Treg subsets in all tested ALPS-Fas patients, relative to CTs (Figure 1A). We then measured the combined intracellular expression of the transcription factors FOXP3 and Helios in CD3^+^CD4^+^-gated T cells, in order to estimate the proportion of Tregs. Although this proportion varied from one patient to another, there was no significant overall difference between patients and CTs. However, when assessing CD25, CD45RA, and CD127 as additional surface markers for Tregs, we observed an abnormally low CD25 expression, an abnormally high CD45RA expression, and a normal CD127 expression in the ALPS-FAS patients (Figure 1B). The low CD25 expression was observed in both FOXP3^+^ and Helios^+^ populations (Figures 1C,D); this is an important observation, since the CD25 is conventionally used as a Treg marker (notably in combination with FOXP3 staining). Furthermore, we observed an abnormally high proportion of naïve Tregs (FOXP3^low^CD45RA^+^) relative to eTregs (FOXP3^high^CD45RA^−^) in the ALPS patients (Figure 1E). This nTreg/eTreg imbalance was not correlated with age (Figure 2A). Similarly, we observed a slightly but significantly elevated proportion of the CD45RA^+^ subset among CD4^+^CD25^−^CD127^+^ Tconvs (Figure 2B). We also investigated the expression of the Treg markers on activated, terminally differentiated and most strongly suppressive FOXP3^high^ eTregs. The latter specifically express the CD15s marker (sialyl Lewis X) recently described in human blood by Miyara et al. (28). We observed a slightly higher proportion of CD4^+^CD127^low^CD15s^+^ cells (Figure 3A) in six out of six ALPS patients tested, relative to CTs. Again, we observed a low expression of the CD25 marker (Figure 3B) and an intermediate level of FOXP3 expression (Figure 3B). By contrast, the levels of Helios expression were very similar in ALPS patients and CTs (Figure 3B). We then analyzed the expression of the CD45RA and CD15s markers on the CD4^+^CD3^+^FOXP3^+^ population in P7 and P14 (Figure 3C, panel 1). The proportion of the CD3^+^CD4^+^CD15s^+^CD45RA^neg^ subpopulation was slightly decreased as compared to controls (Figure 3C, panel 2). Additional markers have been studied on CD4^+^CD127^low^CD15s^+^ cells. We observed increased CCR7 and PD1 staining in three ALPS patients as compared to healthy controls (CTs) (Figure S1 in Supplementary Material). The other markers such as CTLA4 are not modified.

### Assessment of Tconv Sensitivity and Treg Function in Samples From ALPS Patients

**Figure 4:**
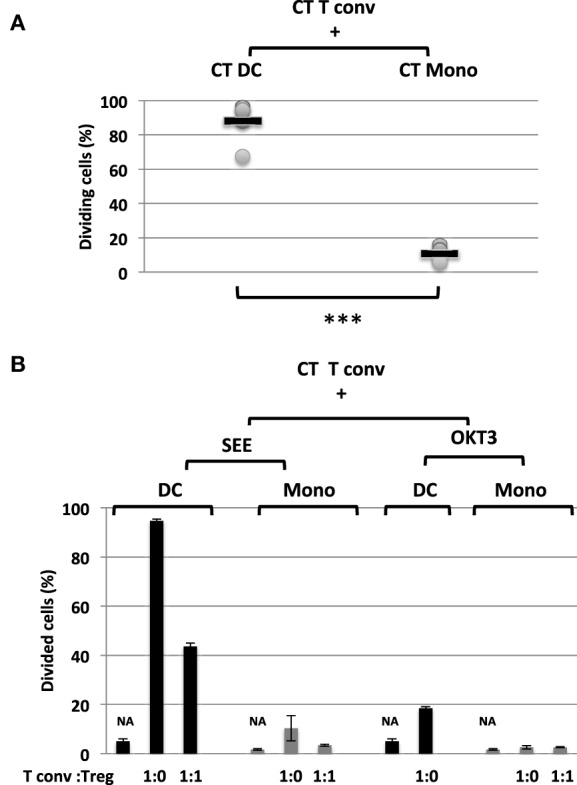
Proportion and function of CD11c^+^CD4^low^ subpopulations **(A)** shows the mean ± SD (range) proportion of proliferating naïve conventional effector T cell (Tconvs) from healthy controls (CTs) (CT Tconv), after carboxyfluorescein succinimidyl ester (CFSE) staining and incubation for 5 days with staphylococcal enterotoxin (SEE) and autologous control dendritic cells (DCs) (CT DC) [*n* = 6; 88.1 ± 12% (67.3–94.7%)] or autologous control monocytes (CT Mono) [*n* = 6; 10.6± 4% (6–16%)]. Statistical analyses were performed using a non-parametric *T*-test (***p < 0.001). **(B)** shows the mean ± SD (range) proportion of proliferating naïve Tconvs from CTs (CT Tconv) after staining with CFSE and incubation for 5 days in the presence of SEE (0.2 ng/ml) or OKT3 (5 µg/ml) with either DC or monocytes. NA = non-activated cells. CT Tregs were added (or not) to give a Tconv:Treg ratio of 0 or 1. The error bars correspond to the mean ± SD for duplicates.

**Figure 5:**
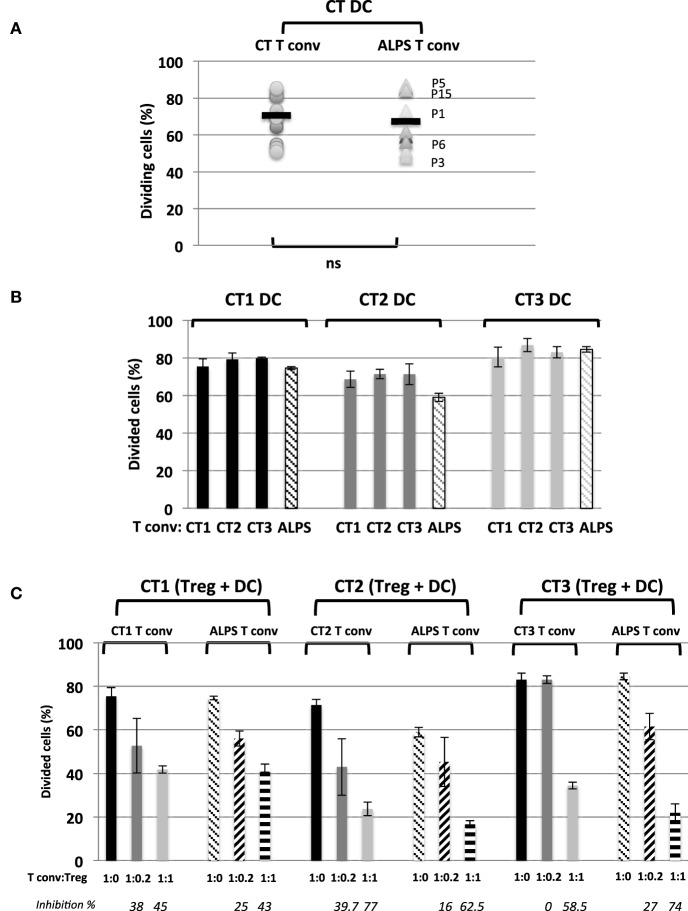
Activation and regulation of naïve conventional effector T cell (Tconvs) from autoimmune lymphoproliferative syndrome (ALPS) patients induced by a CD4^low^ CD11c^+^CD14^−^ dendritic cell (DC) subpopulation from three healthy controls (CTs). **(A)** shows the proportion of proliferating naïve Tconvs from health controls (CTs) or from ALPS patients (ALPS Tconvs) incubated with staphylococcal enterotoxin E (SEE) and DCs from CTs (CT DC). The mean ± SD (range) proportion of CT Tconvs activated by CT DCs was 70 ± 12% (51–85.6%) (*n* = 13) and the mean ± SD (range) proportion of ALPS Tconvs activated by CT DCs was 67.4 ± 15.3% (48.3–87%) (*n* = 9). **(B)** shows the proportion of proliferating naïve Tconvs from three CTs (CT1, CT2, and CT3) and staining with carboxyfluorescein succinimidyl ester (CFSE) and incubation for 5 days with SEE and either DCs from three different CTs (CT1, CT2, and CT3) or DCs from ALPS patients. **(C)** shows the results of the *in vitro* suppression assay using total Tregs isolated from three CTs (CT1, CT2, and CT3) with a Tconv:Treg ratio of 1:1 or 1:0.2 and CFSE-stained naïve Tconvs from three CTs (CT1, CT2, and CT3) or ALPS patients (ALPS Tconvs). The assays were performed with SEE and DCs from the three CTs (CT1, CT2, and CT3) incubated for 5 days. The results are expressed in percentage of proliferating naïve Tconvs. The error bars correspond to the mean ± SD for duplicates. The percentage inhibition of proliferation is indicated in italics. Statistical analyses were performed using a non-parametric Mann–Whitney *U*-test (ns: not significant).

**Figure 6:**
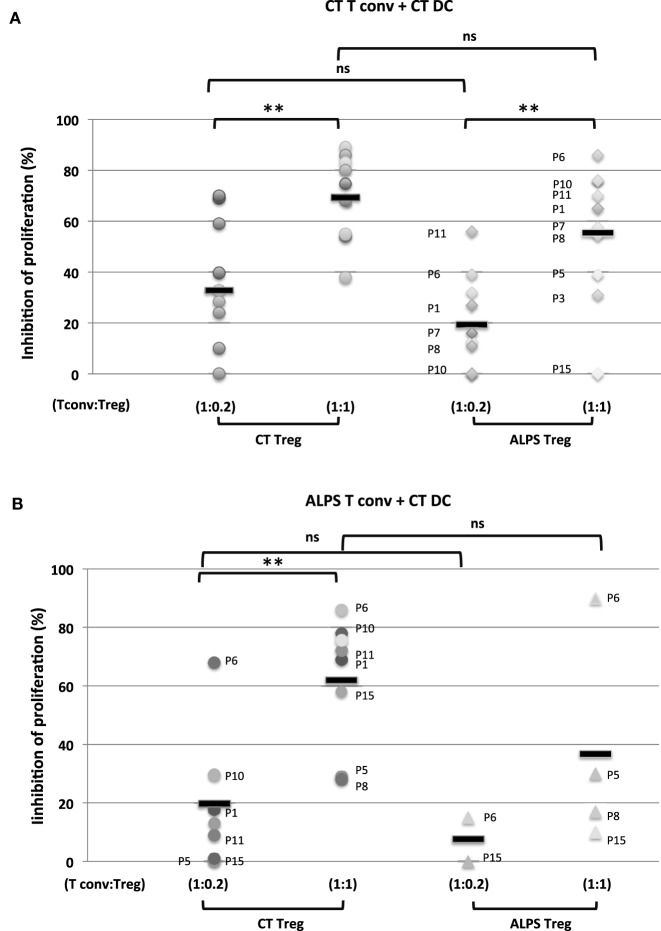
Functional characteristics of regulatory T cells (Tregs) and conventional effector T cell (Tconvs) from autoimmune lymphoproliferative syndrome (ALPS) patients. The figure shows the results of an *in vitro* suppression assay using total Tregs isolated from healthy controls (CTs) (CT Treg) and ALPS patients (ALPS Treg) with a Tconv:Treg ratio of 1:1 or 1:0.2 and carboxyfluorescein succinimidyl ester (CFSE)-stained naïve Tconvs from CTs [CT Tconv: in **(A)**] or ALPS patients [ALPS Tconv, in **(B)**]. The assays were performed with staphylococcal enterotoxin E (SEE) and dendritic cells (DCs) from CTs (CT DC) for 5 days. The results are expressed in percentage of inhibition of proliferating cells. Statistical analyses were performed using a non-parametric *T*-test (***p < 0.001, **p < 0.005, *p < 0.05, ns: not significant). The error bars correspond to the mean ± SD for duplicates. Panel **(A)** shows the mean (range) percentage of inhibition of CT Tconv proliferation by the CT Tregs (69.4 ± 16.4%) (38–89%) for a Tconv:CT Treg ratio of 1:1 (*n* = 10); 32.8 ± 21.6 (0–69%) for a CT Tconv:CT Treg ratio of 1:0.2 (*n* = 10) and by the ALPS Tregs [55.5 ± 26% (0–86%)] for a CT Tconv:ALPS Treg ratio of 1:1 (*n* = 10); 19.4 ± 18.8 (0–56%) for a CT Tconv:ALPS Treg ratio of 1:0.2 (*n* = 10). Panel **(B)** shows the mean (range) percentage of inhibition of ALPS Tconv proliferation by CT Tregs [61.6 ± 21% (28–86%)] for a ALPS Tconv:CT Treg ratio of 1:1 (*n* = 9): 21.6 ± 22% (0–69%) for a ALPS Tconv:CT Treg ratio of 1:0.2 (*n* = 8) or by ALPS Tregs (36.7 ± 36%) (10–90%) for an ALPS Tconv:ALPS Treg ratio of 1:1 (*n* = 4); 7.4 ± 10.6 (0–15%) for an ALPS Tconv:ALPS Treg ratio of 1:0.2 (*n* = 2).

**Figure 7:**
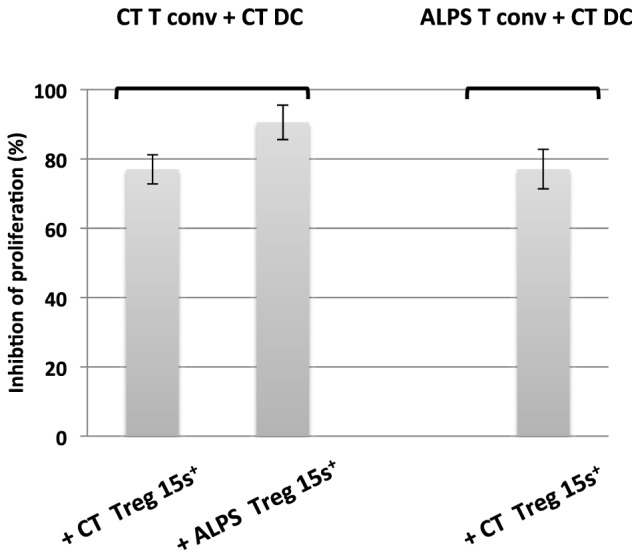
Functional characteristics of CD15s^+^ regulator T cells from autoimmune lymphoproliferative syndrome (ALPS) patients. The figure shows the results of an *in vitro* suppression assay by CD15s^+^ regulatory T cells (Tregs) isolated from two healthy controls (CTs) (CT Treg15S^+^) and two ALPS patients (ALPS Treg15S^+^), with a conventional effector T cell (Tconv):Treg ratio of 1:1 and carboxyfluorescein succinimidyl ester-stained naïve Tconvs from CTs (CT Tconvs) or from ALPS patients (ALPS Tconvs). The assays were performed with staphylococcal enterotoxin E (SEE) and dendritic cells (DCs) from CTs (CT DC) incubated for 5 days. The results are expressed in percentage of inhibition of proliferating cells. The error bars correspond to the mean ± SD for duplicates.

We next investigated the Tregs’ suppressive function on T cell proliferation with regard to naïve CD4^+^CD45RA^+^CD25^-^CD127^+^ Tconvs. The Tregs were stimulated for 5 days with SEE or anti-CD3 antibody (OKT3) in the presence of CD11c^+^CD4^low^ APCs. As previously described (29), we found that DCs (i) were more efficient as APCs than monocytes (Figure S2 in Supplementary Material) and (ii) sustained the proliferation of naïve Tconvs (Figure 4A). Furthermore, stimulation with SEE was stronger than stimulator with OKT3 (Figure 4B). The proliferation of naïve Tconvs was of the same magnitude for ALPS patients and CTs (Figure 5A). Moreover, the naïve Tconvs from patients were stimulated to a similar extent by DCs from three independent CTs—thus emphasizing the assay’s reproducibility (Figure 5B). Furthermore, we observed that the proliferation of Tconvs from these three independent CTs was inhibited to a similar extent, which thus ruled out donor-related variations (Figure 5C). Likewise, the proliferation of Tconvs from ALPS patients was fully inhibited by Tregs from three independent CTs (Figure 5C). Furthermore, Tregs from patients and CTs exhibited similar levels of suppressive activity under autologous and heterologous conditions (Figure 6). Similarly, the CD4^+^CD127^low^CD25^+^CD15s^+^ Tregs isolated from two ALPS patients exhibited the same level of suppressive activity as Tregs from CTs (Figure 7).

## Discussion

The role of FOXP3^+^ Tregs is critical for controlling organ-specific autoimmunity, as demonstrated by the early onset of autoimmune enteropathy and endocrinopathy in IPEX patients and in mice harboring FOXP3 mutations. The extrinsic lymphocyte apoptosis mediated by the death receptor FAS also constitutes a tolerance checkpoint. Indeed, patients or mice harboring an FAS deficiency develop an early-onset lymphoproliferative disease with autoimmunity (essentially against platelets and red cells). However, these two key tolerance checkpoints are unable to compensate for each other, since (i) FAS-mediated apoptosis does not control the autoimmunity that develops in FOXP3-deficient patients and (ii) Tregs do not control the autoimmunity observed in FAS-deficient patients. One can assume that in ALPS, some FAS-deficient target cells (i.e., gut epithelial cells and Langerhans beta cells in the pancreas) are more resistant to destruction by autoreactive T cells. This hypothesis is supported by the less severe autoimmune symptoms observed in FAS- and Treg-deficient mice (30). Moreover, Treg dysfunction might account for the active proliferation seen in ALPS-FAS patients, since impaired FAS-mediated cell death might not fully account for the generalized lymphoproliferation observed in patients and murine models. Hence, we decided to assess the Treg phenotype in ALPS-FAS patients. We systematically observed low expression of the IL-2Ra chain (CD25), which was correlated with low proportions of CD3^+^CD4^+^CD25^high^FOXP3^+^ Treg subsets. This result is consistent with the recently described role of IL-2R signaling in FOXP3 expression (31). Furthermore, we observed a low proportion of CD3^+^CD4^+^CD25^high^CD127^low^ cells in ALPS patients. These Treg subpopulations displayed a naïve phenotype, as confirmed by the high proportion of CD3^+^CD4^+^FOXP3^low^CD45RA^+^ cells. Our results indicate the possible presence of a memory/activated Treg unbalance in ALPS-FAS patients. This abnormal phenotype was not related to previous treatments or age, since it was also observed in untreated patients and in treated patients of various ages. In order to better define the Treg phenotype in ALPS patients, we examined the expression of CD15s—a recently described marker of activated, terminally differentiated, highly suppressive CD15s^+^ CD45RA^−^ eTregs (28). A slight elevation of the CD4^+^CD127^low^CD15s^+^ subpopulation was observed in the ALPS-FAS patients, relative to CTs. However, this subpopulation also displayed a naïve phenotype and expressed normal levels of Helios, intermediate levels of FOXP3 and no CD25—thus defining a particular nTreg subpopulation (CD15s^+^CD25^−^CD127^low^CD45RA^+^Helios^+^FOX^med^) in ALPS-FAS patients. This subpopulation might originate from thymus-derived CD45RA^+^FOXP3^low^Helios^+^CD4^+^ nTregs. Next, we investigated the functional activity of Tregs from ALPS patients by assessing the suppressive function of Tregs on Tconv cell proliferation. Overall, the conventional CD25^+^CD127^low^ Treg subpopulation and the CD127^low^CD15S^+^CD25^low^ Treg subpopulations had similar suppressive functions in samples derived from ALPS-FAS patients and CTs. Moreover, control Tregs (CD25^+^CD127^low^ or CD127^low^CD15S^+^) normally suppressed the ALPS-FAS patients’ Tconvs proliferation. During the test, we have also observed that Treg proliferate (data not shown). This finding indicates that FAS-mediated cell death is not involved in the inhibition of Tconv proliferation by Tregs, or that a redundant pathway is involved. Taken as a whole, the results of our *in vitro* experiments evidenced normal Treg-suppressive function on T cell proliferation, even though the phenotype was abnormal (with a higher proportion of a CD25^low^ Tregs) in the ALPS-FAS patients. The low expression of membrane CD25 by CD4^+^FOXP3^+^ or CD127^low^ or CD15s^+^ populations might be related to the high plasma levels of the soluble form of CD25 (sCD25) detected in the six ALPS-FAS patients tested. This might result from an increased cleavage of CD25 by metalloproteinases, as has been observed in some lymphoproliferative disorders (32, 33). One cannot rule out the possibility that the high proportion of naïve Tregs reflects a reduced activation of the Tregs subset *in vivo* or results from the presence of CD4^+^ terminally differentiated effector CD45RA^+^ T cells (TEMRA), but the fact that these CD4^+^FOXP3^low^CD45RA^+^ cells also express CCR7 and CD27 argues against the possibility that these cells are TEMRAs. In addition, the normal suppressive function observed *in vitro* and the lack of clinical symptoms usually observed in Treg-deficient patients (i.e., autoimmune enteropathy and endocrinopathy) support the hypothesis whereby Treg functions, in particular inhibition of Tconvs proliferation, are unaffected in patients with FAS deficiency—despite the abnormal phenotype observed for circulating cells. However, Tregs from ALPS have been activated with control APCs (because insufficient numbers of ALPS-APCs retrieved upon cell sorting). We thus cannot exclude a putative *in vivo* defect of ALPS Tregs on ALPS-APCs regarding cytokine production (such as IL-10) or modification of some surface molecules on ALPS-APCs (such as CD86). Nevertheless, ALPS patients do not present with clinical features observed in “Tregopathies” (i.e., FOXP3 or CTLA-4 deficiencies) such as lymphocytic tissue infiltrations and organ-specific autoimmunity, in particular autoimmune enteropathy.

In conclusion, our present results show that abnormal proportions of Treg subsets are not necessarily pathogenic features in ALPS. Despite an apparently abnormal Treg phenotype in ALPS-FAS patients, a regulatory defect is unlikely to account for the active lymphoproliferation observed in this disease. Thus, it is still not clear why Tregs cannot control the lymphoproliferation of FAS-deficient T cells and thus cannot prevent autoimmune cytopenia in ALPS patients. Better knowledge of which T cells and/or which types of stimulation are most strongly controlled by the Treg-associated apoptosis pathway may help to identify more specific therapeutic targets in the future.

## Ethics Statement

All patients are registered in the French national primary immunodeficiency database (CEREDIH, Paris, France). The study was performed in accordance with the precepts of the Declaration of Helsinki.

## Author Contributions

FM carried out all the functional experiments and wrote the paper. M-CS carried out the FACS experiments. OP has carried out the cell sorting on ARIA. CP, BN, and AF executed the followup of patients and manuscript discussion. AM-C did the manuscript discussion, and FR-L was responsible for the research project.

## Conflict of Interest Statement

The authors declare that the research was conducted in the absence of any commercial or financial relationships that could be construed as a potential conflict of interest.

## Supplementary Material

Figure S1 Phenotypic characteristics of regulatory T cells (Tregs) in autoimmune lymphoproliferative syndrome (ALPS) patients. CD3^+^CD4^+^-gated T lymphocytes isolated from three healthy controls (CTs) (CT) or three ALPS patients (ALPS: P7, P8, and P15) were stained for CD25 and CD127 **(A)**. Next, CD4^+^CD127^−^CD25^+^-gated T cell subpopulations were stained for CTLA4. In parallel, for two of these patients (ALPS: P7 and P15), CD3^+^CD4^+^-gated T lymphocytes isolated were also stained for CD15s and CD127. Next, CD4^+^CD127^−^CD15s^+^-gated T cell subpopulations were stained for CTLA-4 **(B)**. The CD4^+^CD127^−^CD15s^+^-gated T cell subpopulations of three other patients were also stained for CCR7, GITR, ICOS, CD39, LAG3, CD59, PD1, CD27, and CD28 **(C)**. The mean intensity of fluorescence was added for the control and the ALPS patients. Histograms are normalized to mode for panel **(C)**.

Figure S2 Different proportion of antigen-presenting cell (APC). The mean proportion of total CD11c^+^CD4^low^ APCs (left panel) and the mean ± SD (range) proportion of CD11c^+^CD4^low^CD14^+^ monocytes [82.9 ± 4% (75–89%); *n* = 10; middle panel] and CD11c^+^CD4^low^CD14^−^ DCs [15.8 ± 5% (9–24%); *n* = 11; right panel] among PBMCs from CTs (CT).

## Abbreviations

ALPS, autoimmune lymphoproliferative syndrome; APC, antigen-presenting cell; CFSE, carboxyfluorescein succinimidyl ester; CT, healthy control; DC, dendritic cell; SEE, staphylococcal enterotoxin E; Treg, regulatory T cell; Tconv, conventional effector T cell.
