# Supplementary figures and images for: Autoimmune Lymphoproliferative Syndrome-FAS Patients Have an Abnormal Regulatory T Cell (Treg) Phenotype but Display Normal Natural Treg-Suppressive Function on T Cell Proliferation
Source: Front Immunol. 2018 Apr 9;9:718. doi: 10.3389/fimmu.2018.00718 (PMC5900038; doi:10.3389/fimmu.2018.00718)

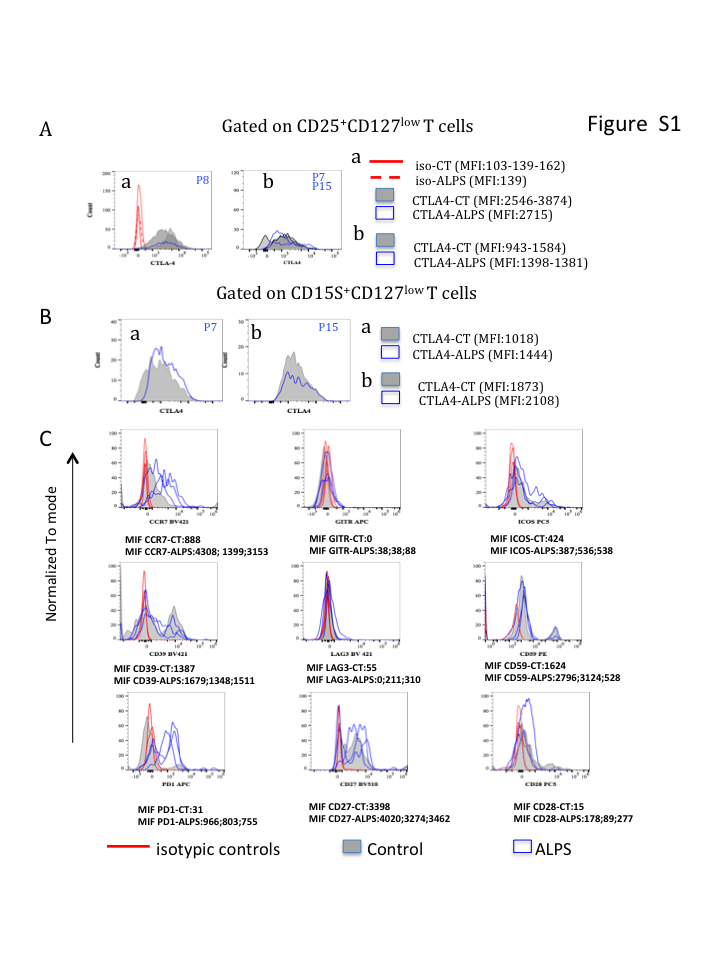

Supplement: Figure S1 — Phenotypic characteristics of regulatory T cells (Tregs) in autoimmune lymphoproliferative syndrome (ALPS) patients. CD3+CD4+-gated T lymphocytes isolated from three healthy controls (CTs) (CT) or three ALPS patients (ALPS: P7, P8, and P15) were stained for CD25 and CD127 (A). Next, CD4+CD127−CD25+-gated T cell subpopulations were stained for CTLA4. In parallel, for two of these patients (ALPS: P7 and P15), CD3+CD4+-gated T lymphocytes isolated were also stained for CD15s and CD127. Next, CD4+CD127−CD15s+-gated T cell subpopulations were stained for CTLA-4 (B). The CD4+CD127−CD15s+-gated T cell subpopulations of three other patients were also stained for CCR7, GITR, ICOS, CD39, LAG3, CD59, PD1, CD27, and CD28 (C). The mean intensity of fluorescence was added for the control and the ALPS patients. Histograms are normalized to mode for panel (C). [file image_1.tiff]

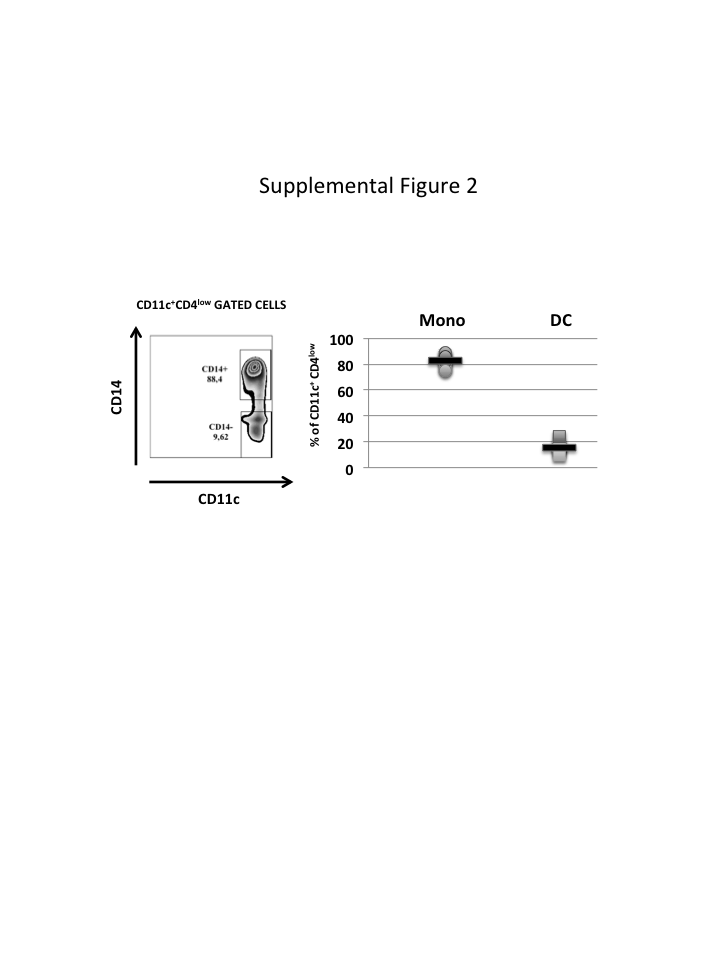

Supplement: Figure S2 — Different proportion of antigen-presenting cell (APC). The mean proportion of total CD11c+CD4low APCs (left panel) and the mean ± SD (range) proportion of CD11c+CD4lowCD14+ monocytes [82.9 ± 4% (75–89%); n = 10; middle panel] and CD11c+CD4lowCD14− DCs [15.8 ± 5% (9–24%); n = 11; right panel] among PBMCs from CTs (CT). [file image_2.tiff]
